# Role of Nitric Oxide Synthases in Early Blood-Brain Barrier Disruption following Transient Focal Cerebral Ischemia

**DOI:** 10.1371/journal.pone.0093134

**Published:** 2014-03-26

**Authors:** Zheng Jiang, Chun Li, Denise M. Arrick, Shu Yang, Alexandra E. Baluna, Hong Sun

**Affiliations:** Department of Cellular Biology & Anatomy, Louisiana State University Health Sciences Center-Shreveport, Shreveport, Louisiana, United States of America; University of Missouri, United States of America

## Abstract

The role of nitric oxide synthases (NOSs) in early blood-brain barrier (BBB) disruption was determined using a new mouse model of transient focal cerebral ischemia. Ischemia was induced by ligating the middle cerebral artery (MCA) at its M2 segment and reperfusion was induced by releasing the ligation. The diameter alteration of the MCA, arterial anastomoses and collateral arteries were imaged and measured in real time. BBB disruption was assessed by Evans Blue (EB) and sodium fluorescein (Na-F) extravasation at 3 hours of reperfusion. The reperfusion produced an extensive vasodilation and a sustained hyperemia. Although expression of NOSs was not altered at 3 hours of reperfusion, L-NAME (a non-specific NOS inhibitor) abolished reperfusion-induced vasodilation/hyperemia and significantly reduced EB and Na-F extravasation. L-NIO (an endothelial NOS (eNOS) inhibitor) significantly attenuated cerebral vasodilation but not BBB disruption, whereas L-NPA and 7-NI (neuronal NOS (nNOS) inhibitors) significantly reduced BBB disruption but not cerebral vasodilation. In contrast, aminoguanidine (AG) (an inducible NOS (iNOS) inhibitor) had less effect on either cerebral vasodilation or BBB disruption. On the other hand, papaverine (PV) not only increased the vasodilation/hyperemia but also significantly reduced BBB disruption. Combined treatment with L-NAME and PV preserved the vasodilation/hyperemia and significantly reduced BBB disruption. Our findings suggest that nNOS may play a major role in early BBB disruption following transient focal cerebral ischemia via a hyperemia-independent mechanism.

## Introduction

Stroke continues to be a leading cause of death and permanent disability worldwide [1]. Due to the use of thrombolytic therapy, transient focal cerebral ischemia has become one of the most common types of ischemic stroke. Although establishment of reperfusion is important for the cells in the penumbral zone, reperfusion is the most powerful independent predictor of BBB disruption [2]. BBB disruption occurs in early and late phases following ischemic stroke [3]. Early BBB disruption can be found as early as within first hour of reperfusion [4], whereas late BBB disruption occurs between 24 hours to 72 hours of reperfusion [3]. Early BBB disruption has been considered as an antecedent event to infarction and hemorrhagic transformation [2], [5]. Although the mechanism remains poorly delineated, activation of matrix metalloproteinases (MMPs) 2 and 9 has been implicated in the pathogenesis of early BBB disruption following transient focal cerebral ischemia [3].

Nitric oxide (NO), synthesized by NO synthases (NOSs), is a well-known vasodilator, neurotransmitter and key mediator of immunity. However, NO has detrimental effect under pathophysiological conditions especially when it is excessively produced and/or oxidative stress is being involved. Overproduction of NO may lead cell damage by directly altering protein structure/function and/or indirectly through the formation of highly reactive peroxynitrite [6], [7]. The rapid restoration of blood flow following ischemia increases the level of tissue oxygenation but accounts for a burst of NO and superoxide generation, which may result in a rapid increase in peroxynitrite formation. Peroxynitrite was reported to activate MMPs 2 and 9 following transient focal cerebral ischemia [8]. A previous study found that preischemic treatment with L-NAME, a non-specific inhibitor of NOS, significantly reduced early BBB disruption following transient global ischemia [9]. Recently, preischemic treatment with L-NAME was shown effective in preventing early BBB disruption following transient focal cerebral ischemia [10]. In addition, methylene blue ameliorated early BBB disruption following transient global ischemia by decreasing NO metabolites [11]. All three isoforms of NOS, endothelial NOS (eNOS), neuronal NOS (nNOS) and inducible NOS (iNOS), may be involved in NO synthesis following transient focal cerebral ischemia. Thus, our first goal was to identify the NOS isoform that plays the major role in early BBB disruption following transient focal cerebral ischemia. Since postischemic hyperemia has been suggested to associate with adverse events, including ischemic edema, BBB disruption, and poorer outcome [12], it is possible that excessive production of NO during reperfusion lead early BBB disruption via a sustained vasodilation/hyperemia. Thus, our second goal was to determine whether postischemic vasodilation/hyperemia is related to early BBB disruption.

## Materials and Methods

### Preparation of animals

Animal studies were approved by the University Committee on Animal Resources of the Louisiana State University Health Science Center-Shreveport and conducted in accordance with the ARRIVE (Animal Research: Reporting In Vivo Experiments) guidelines for the care and use of laboratory animals. At 4 months of age (body weight 25 to 30 g), male C57BL/6J mice (n = 72) were anesthetized with thiobutabarbital sodium (Inactin, 100 mg/kg, ip), and a tracheotomy was performed. The mice were ventilated mechanically with room air and supplemental oxygen using a small animal ventilator (Harvard apparatus, March, Germany) at a fixed inhalation-exhalation ratio (1∶1). A catheter was placed into right femoral vein for injection of Evans Blue (EB) and sodium fluorescein (Na-F). Blood pressure was measured using a CODA mouse tail-cuff system (Kent Scientific, Torrington, CT, USA). Body temperature was maintained at 37°C using a rectal temperature regulated heating pad (TC1000, CWE, Ardmore, PA, USA).

**Figure 1:**
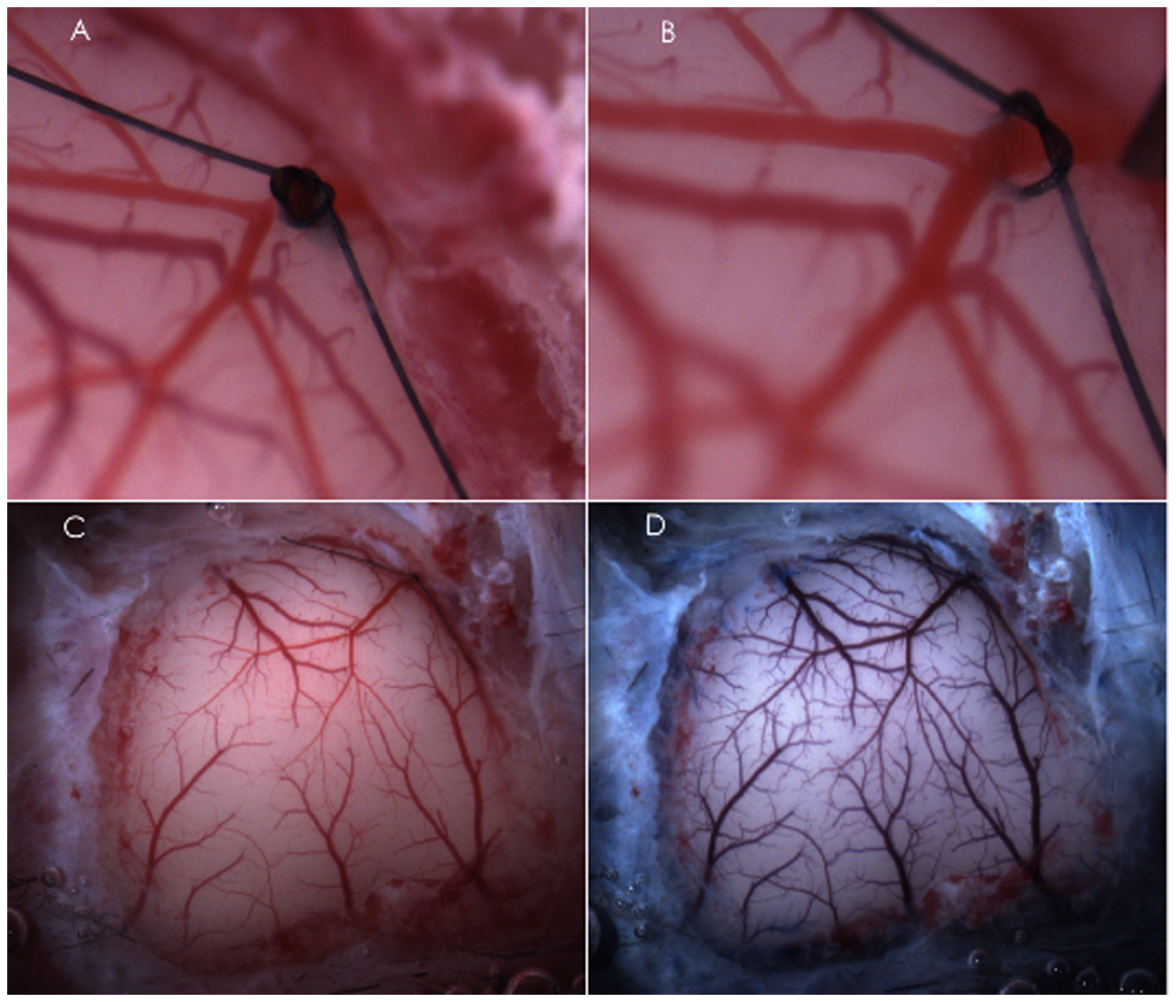
Representative cranial windows showing MCAL/reperfusion induced by ligating (A) and releasing (B) the MCA at its M2 segment and the surface vasculature of frontal, parietal and temporal cortex before (C) and after (D) injection of EB and Na-F.

To perform ischemia/reperfusion, observe cerebral vasculature and topically administrate NOS inhibitors and NO-independent vasodilator, the mice were placed on a stereotaxic frame. A cranial window (6 mm×8 mm, 1 mm from midline to the zygomatic arch) was prepared over the left frontal, parietal and temporal cortex (Figure 1). The cranial window was suffused with artificial cerebrospinal fluid bubbled continuously with 95% nitrogen and 5% carbon dioxide. The temperature of the suffusate was maintained at 37°C. The cranial window was connected via a three-way valve to an infusion pump, which allowed for infusion of NOS inhibitors and NO-independent vasodilator into the cranial window. This method will maintain temperature, pH, PCO_2_ and PO_2_ of the cranial window at normal physiological range during the experiment. The MCA was ligated at its M2 segment just proximal to the first bifurcation/trifurcation with a 10-0 nylon suture for 2 hours (Figure 1). To prevent potential damage to the MCA during the ligation and easily induce reperfusion, a segment of 5-0 monofilament nylon suture was ligated together with the MCA. Reperfusion was induced by removing the nylon suture at 2 hours of ischemia (Figure 1). In Sham group, 10-0 nylon suture was not ligated.

### Experimental protocol

The cranial window was superfused with artificial cerebrospinal fluid for 30 minutes before ligating the MCA. We examined the influences of NOS inhibitors (non-specific NOS inhibitor: L-nitro-arginine methyl ester (L-NAME); selective nNOS inhibitors: N(omega)-propyl-L-arginine (L-NPA) and 7-nitroindazole (7-NI); relatively selective eNOS inhibitor: L-N(5)-(1-iminoethyl)ornithine (L-NIO); selective iNOS inhibitor: aminoguanidine (AG)) and NO-independent vasodilator (papaverine (PV)) on MCA ligation (MCAL)/reperfusion-induced regional vasodilation, hyperemia and early BBB disruption. The animals were divided into seven groups: Control (n = 12), Sham (n = 4), L-NAME (n = 8), L-NPA (n = 8), 7-NI (n = 8), L-NIO (n = 8), AG (n = 8), PV (n = 8) and L-NAME+PV (n = 8). Drugs were mixed in artificial cerebrospinal fluid and then superfused into the cranial window. The superfusion of NOS inhibitors and NO-independent vasodilator was started 10 minutes prior to the reperfusion and then continued throughout 3-hour reperfusion. The window concentration of L-NAME (300 µM), L-NPA (5 µM), 7-NI (10 µM), AG (10 mM) and PV (10 µM) are reported effective in cerebral vasculature [13], [14], [15], [16], [17].

### Measurement of regional vasodilation

Image recording of cerebral vasculature was started one minute before MCAL and continued throughout the experiment with an Orca-Flash 2.8 CMOS camera (Szxz-FOF, Olympus, Japan). The images at 5, 10, 30, 60, 90, and 120 minutes of ischemia and 5, 10, 30, 60, 90, 120, 150, and 180 minutes of reperfusion were analyzed with a Visiopharm Integrator System (Olympus, Japan). Percentage change of vascular diameter at the middle of the MCA branches, arterial anastomoses, and terminal branches of the anterior cerebral artery (ACA) and posterior cerebral artery (PCA), which are anastomosing the terminal branches of the MCA, were calculated. We classified all the imaged vessels by their baseline diameters: MCA: M3 (d≥70 µm), M3 (60 µm≤d<70 µm), M3 (50 µm≤d<60 µm), M3 (40 µm≤d<50 µm), M3 (30 µm≤d<40 µm), M3 (d<30 µm); arterial anastomoses and terminal branches of ACA/PCA.

### Measurement of regional cerebral blood flow (rCBF)

To determine whether cerebral vasodilation during ischemia produces a hyperemia, we monitored rCBF at left somatosensory area. The probe of Laser Doppler Flowmetry (Periflux System 5000, Perimed, Sweden) was attached on the surface of anterior central gyrus 4–5 mm lateral to the midline. The rCBF was measured at the time points when cerebral vasculature was imaged. Changes in rCBF is calculated and expressed as percentage changes to the baseline.

### Assessment of BBB disruption

EB and Na-F were used to evaluate large and small solute permeability of BBB respectively. EB (4%, 6 ml/kg, Sigma) and Na-F (0.4%, 6 ml/kg, Sigma) saline solution were mixed and injected into the femoral vein at two and half hours of reperfusion (Figure 1 D). At 3 hours of reperfusion, mice were transcardially punctured to collect blood sample and then perfuse saline (about 50 ml) until colorless fluid was obtained from the right atrium. Brains were removed quickly, frozen in liquid nitrogen and stored at −80°C. To determine the content of EB and Na-F in the brain tissues, ischemic side and contralateral cerebral hemispheres were separated, homogenized in 400 µl PBS and centrifuged at 4°C for 10 minutes at 1300 g. The supernatant was collected and protein concentration was determined by the Bradford method (Bio-Rad, CA, USA) with BSA as the standard. The supernatant was added with 50% trichloroacetic acid at 2∶1 ratio and stored at 4°C for 12 hours. The mixture was centrifuged at 4°C for 15 minutes at 15000 g, 100 µl supernatant was added with Borate buffer at 1∶2 ratio and transferred to 96-well black plates. The concentration of Na-F was determined at 485-nm excitation/538-nm emission with Cyto Fluor Series 4000 fluorescence multiwell plate reader (PerSeptive Biosystems, MA, USA). To determine the content of EB in the brain tissues, the pellet of the homogenates were suspended with 500 µl formamide and incubated at 50°C for 72 hours. The mixture was centrifuged at 4°C for 20 minutes at 21000 g, and 300 µl supernatant was transferred to 96-well clear plate. The concentration of EB was determined at 550-nm excitation/620-nm emission with a Microplate Spectrophotometer, Spectra Max 190 (Molecular Devices, CA, USA). To normalize the content of EB and Na-F in brain tissues, plasma concentration of EB and Na-F was measured. Blood sample was centrifuged at 4°C for 10 minutes at 3000 g. The supernatant was diluted with water at 1∶4 ratio. Diluted plasma was added with 20% trichloroacetic acid at 1∶10 ratio and put into cool room (4°C) for 12 hours. The mixture was centrifuged at 4°C for 15 minutes at 15000 g, 100 µl supernatant was added with Borate buffer at 1∶2 ratio and transferred to 96-well black plates. The fluorescence of Na-F was read at 485-nm excitation/538-nm emission. To determine the content of EB in the blood samples, 25 µl 1∶4 diluted plasma was suspended with 400 µl formamide and incubated at 50°C for 72 hours. The mixture was centrifugation at 4°C for 20 minutes at 21000 g, and 30 µl supernatant was transferred to 96-well clear plate with 270 µl water for further dilution. The concentration of EB was read at 550-nm excitation/620-nm emission. The tissue content of EB and Na-F in ischemic side was normalized by its concentration in plasma and protein concentration of the homogenate and finally expressed as a ratio to its concentration in contralateral cerebral hemisphere.

### Western Blot Analysis

Additional four brains from the control group were used to measure the expression of NOSs. Under microscope, infarct core was identified as opaque area, and the cortex bordering 2 mm the infarct core was considered as the peri-infarct area. Parietal cortex and striatum tissues punched at the peri-infarct and contralateral corresponding areas were used for measuring eNOS, nNOS and iNOS. Brain tissues were homogenized in ice-cold lysis buffer containing 150 mM NaCl, 50 mM Tris HCl, 10 mM EDTA, 0.1% Tween-20, 1% Triton, 0.1% mercaptoethanol, 0.1 mM phenylmethylsulfonylfluoride, 5 µg/mL leupeptin, and 5 µg/mL aprotinin, pH 7.4. Homogenates were centrifuged at 4°C for 10 minutes at 10000 g, and the supernatants were collected. Protein concentration was determined by the Bradford method (Bio-Rad, CA, USA) with BSA as the standard. SDS polyacrylamide gel electrophoresis (SDS-PAGE) was performed on a 10% gel on which 20 µg of total protein per well was loaded. After SDS-PAGE, the proteins were transferred onto polyvinylidene difluoride membrane. Immunoblotting was performed with the use of mouse anti-eNOS (BD Bioscience, CA, USA), mouse anti-nNOS (Santa Cruz, TX, USA), rabbit anti-iNOS (Santa Cruz, TX, USA) and rabbit anti-β-tubulin (Santa Cruz, TX, USA) as primary and peroxidase conjugated goat anti-mouse IgG and goat anti-rabbit IgG as the second antibodies. The bound antibody was detected by enhanced chemiluminescence (ECL) detection (Pierce Chemical, IL) and the bands were analyzed using ChemiDoc™ MP Imaging System (Bio-Rad). For quantification, NOS proteins were normalized to the expressed β-tubulin.

### Statistical Analysis

For comparison of the various treatments, results were compared using a two-way repeated measure ANOVA with Tukey's post hoc test. Student *t* tests were used to compare NOS expression before and following transient focal cerebral ischemia. Values are means ± SEM. A p value of 0.05 or less was considered to be significant.

## Results

### MCAL/reperfusion-induced cerebral vasodilation

**Figure 2:**
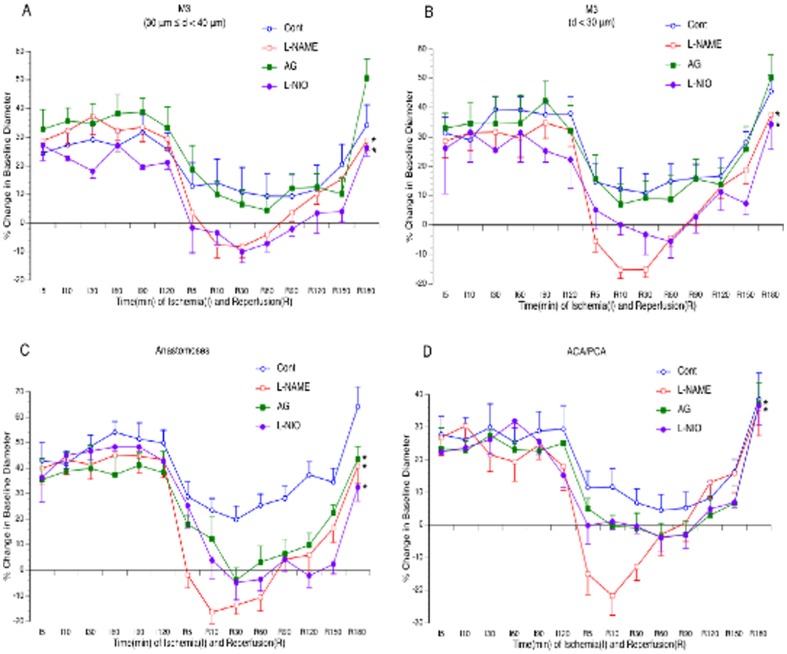
Effects of topical treatment with L-NAME, L-NIO and AG on vasodilation of the MCA (A–B), arterial anastomoses (C) and terminal branches of the ACA/PCA (D) during reperfusion. Values are means ± SE for 8 mice in each group. *P<0.05 vs. Control.

Cerebral vasodilation occurred in nearly all branches of MCA, arterial anastomoses, and terminal branches of ACA/PCA during both ischemia and reperfusion. It started immediately after MCAL, reached to the peak between 10 to 30 minutes after MCAL and kept at a steady state during the rest period of ischemia. The magnitude of cerebral vasodilation was varied in branches more than 40 µm in diameter and similar in smaller branches (Figure 2). Cerebral vasodilation continued during the entire 3-hour reperfusion. The magnitude of cerebral vasodilation during reperfusion was less during the first 150 minutes and greater at 180 minutes of reperfusion compared with the vasodilation during ischemia.

### Effects of NOS inhibitors on cerebral vasodilation

**Figure 3:**
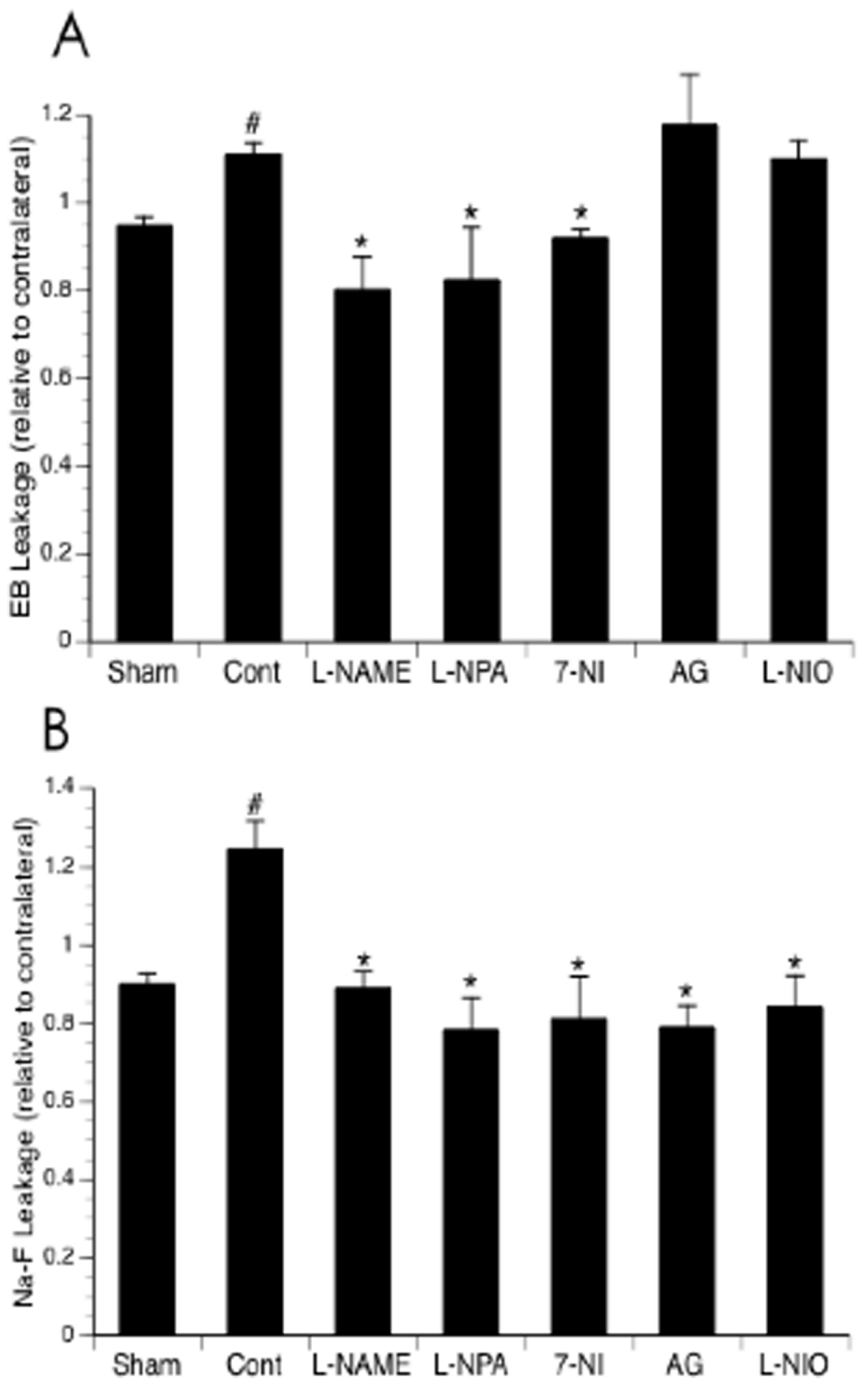
Effects of topical treatment with L-NPA and 7-NI on vasodilation of the MCA (A–B), arterial anastomoses (C) and terminal branches of the ACA/PCA (D) during reperfusion. Values are means ± SE for 8 mice in each group. *P<0.05 vs. Control.

As shown in Figure 2, topical treatment with 300 µM L-NAME abolished cerebral vasodilation in all branches of the MCA, arterial anastomoses and terminal branches of ACA/PCA during reperfusion. Topical treatment with L-NIO at 10 µM similarly inhibited reperfusion-induced cerebral vasodilation as L-NAME. In contrast, topical treatment with 10 mM AG (Figure 2), 5 µM L-NPA and 10 µM 7-NI (Figure 3) only significantly attenuated the vasodilation in arterial anastomoses.

### Effects of NOS inhibitors on BBB disruption

**Figure 4:**
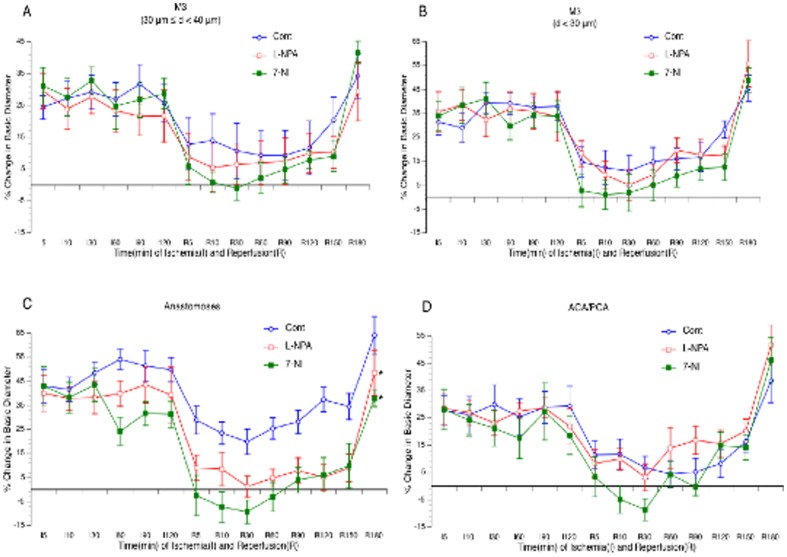
Effects of topical treatment with L-NAME, L-NIO, L-NPA, 7-NI and AG on EB (A) and Na-F (B) extravasation at 3 hours of reperfusion. Values are means ± SE for 8 mice in each group. *P<0.05 vs. Control.

Since the ligation of the MCA in our new model was complete with little individual variability, the extravasation of EB and Na-F was steady and significant at 3 hours of reperfusion in mice undergone MCAL compared with the mice in Sham group (Figure 4). Topical treatment with L-NAME completely prevented both EB and Na-F extravasations at 3 hours of reperfusion. Either L-NPA or 7-NI similarly inhibited both EB and Na-F extravasations as L-NAME. In contrast, topical treatment with L-NIO and AG only inhibited Na-F extravasation (Figure 4).

### Effects of PV on vasodilation/hyperemia and BBB disruption

**Figure 5:**
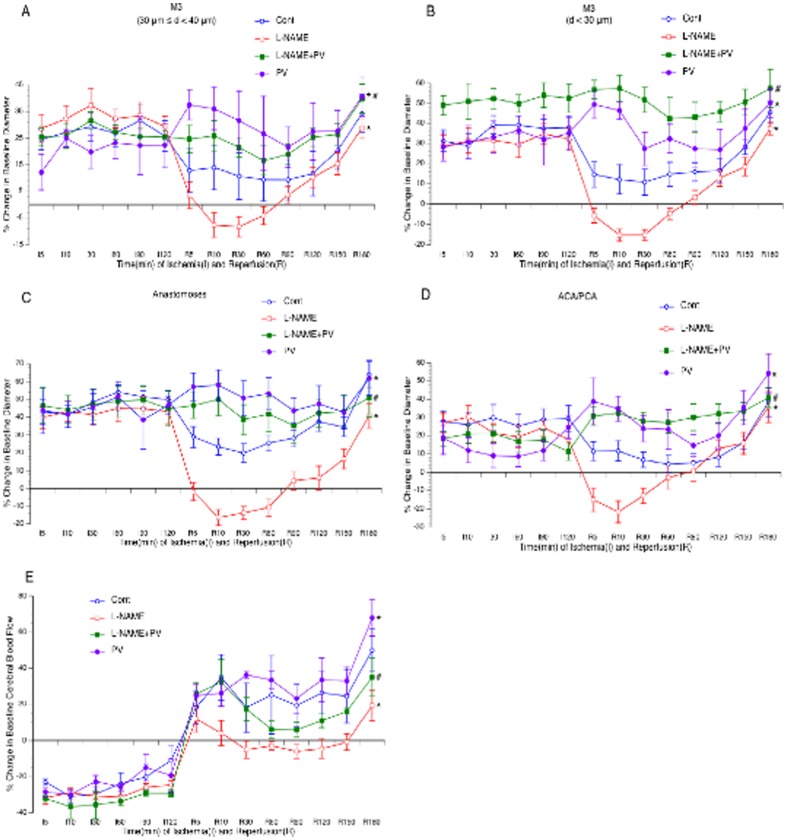
Effects of topical treatment with PV and L-NAME on vasodilation of the MCA (A–B), arterial anastomoses (C) and terminal branches of the ACA/PCA (D) and CBF of somatosensory area (E) during reperfusion. Values are means ± SE for 8 mice in each group. *P<0.05 vs. Control. #P<0.05 vs. L-NAME.

**Figure 6:**
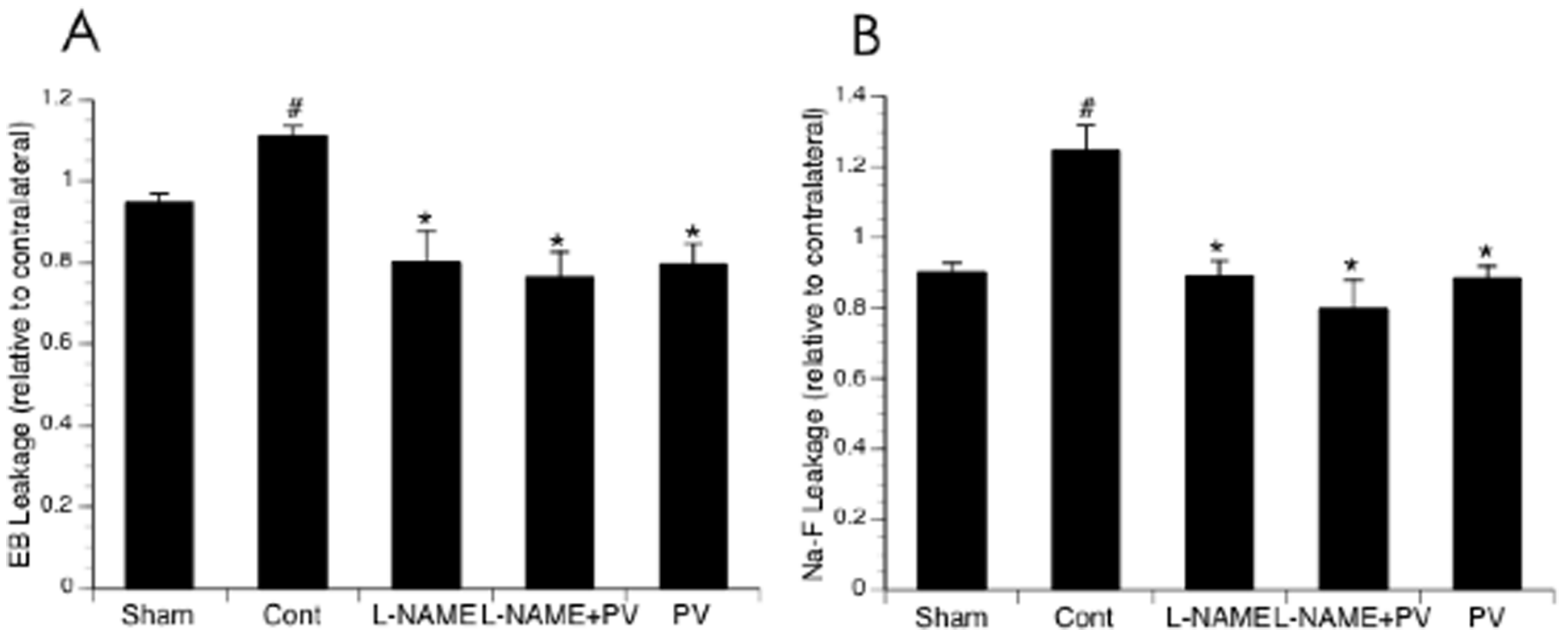
Effects of topical treatment with PV and L-NAME on EB (A) and Na-F (B) extravasation at 3 hours of reperfusion. Values are means ± SE for 8 mice in each group. *P<0.05 vs. Control.

As shown in Figure 5, topical treatment with 10 µM PV alone further induced cerebral vasodilation and hyperemia during reperfusion. Interestingly, PV significantly reduced both EB and Na-F extravasations at 3 hours of reperfusion (Figure 6). In addition, PV abolished the inhibitory effect of L-NAME on cerebral vasodilation and hyperemia during reperfusion (Figure 5), but didn't affect the inhibitory effect of L-NAME on EB and Na-F extravasations (Figure 6).

### NOS expression following 2-hourMCAL/3-hour reperfusion

**Figure 7:**
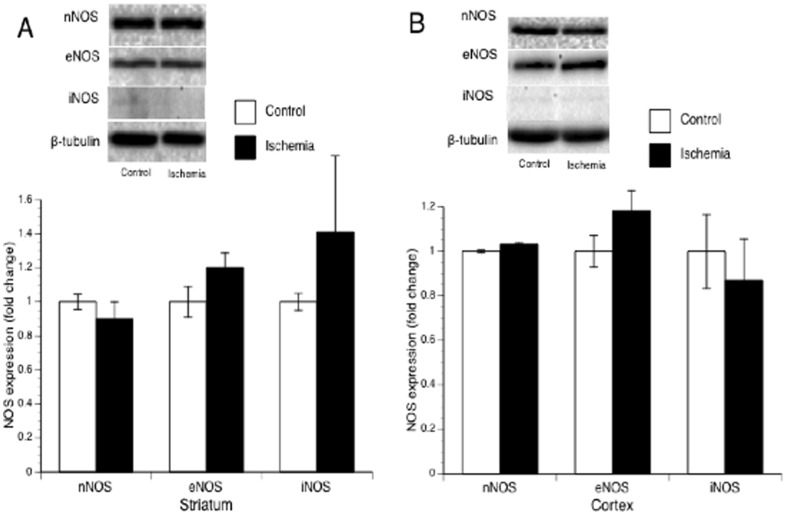
Protein expression of nNOS and eNOS in striatum (A) and parietal cortex (B) punched at the peri-infarct area and the contralateral corresponding areas at 3 hours of reperfusion.

To determine whether postischemic hyperemia and early BBB disruption is related to an upregulated NOS expression, we measured the protein expression of nNOS, eNOS and iNOS in parietal cortex and striatum punched at the peri-infarct area. However, all three isoforms of NOS were not altered in either parietal cortex or striatum at 3 hours of reperfusion (Figure 7).

## Discussion

The present study developed a new transient focal cerebral ischemia model to observe cerebral vasculature, measure CBF and study BBB permeability. In addition, there are several new findings from this study. First, reperfusion following focal cerebral ischemia produced a sustained NOS-dependent vasodilation and hyperemia, in which all three types of NOS might be involved. Second, nNOS inhibitors have strong inhibitory effect on the early BBB disruption. Third, early BBB disruption following transient focal cerebral ischemia was not related to the sustained vasodilation and hyperemia. Fourth, PV significantly suppressed early BBB disruption following transient focal cerebral ischemia. We suggest that nNOS may play a major role in early BBB disruption following transient focal cerebral ischemia via a hyperemia-independent mechanism. In addition, NO-independent vasodilators may be beneficial in preventing early BBB disruption following ischemic stroke.

A reliable animal model of transient focal cerebral ischemia is critical to study the pathophysiology of ischemic stroke and evaluate therapeutic approaches. Intraluminal occlusion of MCA using a suture methodology has been extensively used due to a minimal direct invasion to the brain [18]. However, the final ischemic brain damage with this method could be variable due to possible incomplete obstruction of CBF to the MCA territory. Suture type, length of insertion and the anatomical variations at the MCA origin are factors that may affect the obstruction of the MCA [19], [20]. Thus, a modified suture technique was suggested to improve the consistency of MCAO model [21]. In present study, focal cerebral ischemia was induced by directly ligating MCA at its M2 segment. This new model has several strengths. First, a complete obstruction of CBF to the MCA territory can be identified under the surgical microscope. Second, the cranial window makes the topical treatment possible, and thus avoids the systemic influence of experimentally tested agonists/antagonists and neuroprotective agents. Third, response of cerebral vasculature during either systemic or topical treatment of the drugs can be measured in real time. However, it is important to note that only cerebral cortex can be studied using this model since ligating the MCA at M2 segment doesn't obstruct the blood flow to striatum. In addition, the cranial window impedes the possibility to study the late ischemia/reperfusion injury.

There are various factors including mechanical factors, circulating stimuli, endothelium-derived vasoactive factors, and perivascular nerves that contribute to the tone of cerebral arteries under basal conditions [22]. When focal ischemia occurs, dilation of the pre-existing collateral arteries is a critical mechanism for regional CBF rebuilding. The present study is the first to directly show that cerebral vasodilation and hyperemia are substantially induced during ischemia and reperfusion. In addition, we found that the vasodilation appeared more prominent in/near the terminal branches of MCA and anastomosing arteries indicating that these arteries may be more critical for effective collateral circulation remodeling. NO is an important signaling molecule that regulates vascular tone [23]. It has been suggested that a burst of NO generation occurs within the first few minutes after ischemia [24], [25] and this ischemia-induced NO overproduction has been correlated with glutamate-mediated increase in intracellular Ca^2+^ concentrations, which subsequently results in a calmodulin-dependent activation of nNOS in the ischemic area [26], [27]. In addition to increased activity, there is also a transient increase in the expression of both nNOS [28] and eNOS [29] in the ischemic area, which may also contribute to the initial burst of NO generation and NO-mediated vasodilation and hyperemia [30]. Following the early and transient rise in NO formation, a secondary wave of NO overproduction starts to develop several hours after the initial ischemic insult and is sustained for up to 4–7 days [31], [32]. This enhanced and prolonged NO release can be entirely ascribed to the induced expression of iNOS in response to locally produced inflammatory cytokines following ischemia with or without reperfusion [33], [34]. However, NO concentration is transiently increased by 50% for about 30 minutes if reperfusion occurs even when iNOS has not been induced [35], [36]. A previous study suggested that NO generation during early reperfusion was mostly derived from eNOS [36]. In the present study, eNOS-specific inhibitor showed a strong inhibitory effect on cerebral vasodilation during early reperfusion, whereas nNOS-specific and iNOS-specific inhibitors only showed inhibitory effect on cerebral dilation in certain branches of the MCA. Thus, although all three isoforms of NOS are involved, eNOS may play a major role in the vasodilation and hyperemia during early reperfusion.

The present study identified the NOS isoform that plays the major role in early BBB disruption following transient focal cerebral ischemia. A recent study reported that preischemic treatment with L-NAME was beneficial in preventing early BBB disruption following transient focal cerebral ischemia [10]. In the present study, L-NAME, topically given at 300 µM during reperfusion, completely inhibited both cerebral vasodilation and early BBB disruption. We further found that topical treatment with L-NPA at 5 µM and 7-NI at 10 µM during reperfusion produced similar inhibitory effect on BBB disruption as L-NAME. In contrast, topical treatment with L-NIO at 10 µM and AG at 10 mM only had inhibitory effect on small solute permeability of BBB. Although we only tested one dose for each specific inhibitor, the dose is not a likely factor to make difference on BBB disruption since L-NIO produced a stronger inhibition on cerebral vasodilation than L-NPA and 7-NI. Moreover, iNOS expression was not upregulated at 3 hours of reperfusion. Thus, nNOS may play a major role in early BBB disruption following transient focal cerebral ischemia. Excessive production of NO during ischemia/reperfusion may lead BBB disruption by altering protein structure/function and/or through the formation of highly reactive peroxynitrite. In the present study, an extensive vasodilation was found in the ligated MCA, arterial anastomoses and collateral arteries during the entire ischemia. In addition, a reversed blood flow from dilated collateral arteries and arterial anastomoses was seen to supply the ligated artery. These findings suggest that vascular endothelial cells may suffer a less hypoxia than neurons and the surrounding support cells during ischemia. Upon reoxygenation, thus, excessive NO and superoxide may be mainly produced by neurons and/or the surrounding support cells.

Hyperemia during reperfusion was reported to associate with adverse events including BBB disruption in rat MCAO model [12]. Since NO is potent vasodilator, excessive production of NO during reperfusion may also lead BBB disruption via a sustained hyperemia. In the present study, however, L-NIO significantly inhibited cerebral vasodilation during reperfusion, but only reduced small solute permeability of BBB. In addition, PV preserved the inhibitory effect of L-NAME on cerebral vasodilation/hyperemia, but did not affect the inhibitory effect of L-NAME on early BBB disruption. These findings suggest that excessive production of NO during reperfusion leads BBB disruption via a vasodilation/hyperemia-independent mechanism. Since both early BBB disruption and postischemic hyperemia associate with an excessive production of NO, it is not surprised to find a correlation between hyperemia and early BBB disruption [12]. Interestingly, we further found a reduced BBB disruption in PV-treated group, in which the hyperemia was actually increased. Thus, hyperemia during early reperfusion may be beneficial on preventing early BBB disruption. The mechanism underlying the beneficial effect of PA is not clear, but may be related to an increased regional CBF, which may improve regional oxidative status. Thus, a combined treatment with antioxidant, nNOS inhibitor and NO-independent vasodilator during early reperfusion may lead to a better prognosis in patients with transient focal cerebral ischemia.

There are several limitations in the present study. First, BBB disruption also occurs between 24 hours to 72 hours of reperfusion. Due to the limitation of the model, late-stage BBB disruption couldn't be evaluated. Second, hyperemia during reperfusion may be beneficial on preventing early BBB disruption. It would be necessary to further study the mechanism. Third, while a lot of people under 65 have strokes, ischemic stroke is common among the elderly people. Cerebral vasoreactivity may be different between young people and elderly people [37], [38]. Since young animals were used in our studies, it would be necessary to further evaluate the effect of NOS inhibitors and NO-independent vasodilators on the BBB disruption in aged animals.

In summary, the present study determined the role of NOSs in early BBB disruption following transient focal cerebral ischemia. We suggest that nNOS-mediated NO may be responsible for the BBB disruption. In addition, NO-independent vasodilator-induced hyperemia may be beneficial on preventing the BBB disruption.
